# Circular retrotransposition products generated by a LINE retrotransposon

**DOI:** 10.1093/nar/gks859

**Published:** 2012-09-12

**Authors:** Jeffrey S. Han, Shirley Shao

**Affiliations:** Department of Embryology, Carnegie Institution for Science, Baltimore, MD 21218, USA

## Abstract

Non-long terminal repeat (non-LTR) retrotransposons are highly abundant elements that are present in chromosomes throughout the eukaryotic domain of life. The long interspersed nuclear element (LINE-1) (L1) clade of non-LTR retrotransposons has been particularly successful in mammals, accounting for 30–40% of human genome sequence. The current model of LINE retrotransposition, target-primed reverse transcription, culminates in a chromosomally integrated end product. Using a budding yeast model of non-LTR retrotransposition, we show that in addition to producing these ‘classical’, chromosomally integrated products, a fungal L1 clade member (Zorro3) can generate abundant, RNA-derived episomal products. Genetic evidence suggests that these products are likely to be formed via a variation of target-primed reverse transcription. These episomal products are a previously unseen alternative fate of LINE retrotransposition, and may represent an unexpected source for *de novo* retrotransposition.

## INTRODUCTION

Non-long terminal repeat (non-LTR) retrotransposons are highly abundant elements that are present in chromosomes throughout the eukaryotic domain of life. Members of the L1 clade of non-LTR retrotransposons are of particular interest, as they have amplified to high copy number in mammalian genomes. Sequencing projects have shown that 15–30% of mammalian genomes comprise long interspersed nuclear element (LINE) sequence (1–8). An additional 8–20% of mammalian sequence is derived from non-LINE sequences (e.g. Alu elements, SVA elements and processed pseudogenes) that parasitize LINE machinery to retrotranspose (9). The process of L1 retrotransposition is still occurring in humans today and is a source of DNA damage (10,11), *de novo* mutations (12,13) and genetic variability (14–20).

**Figure 1.**
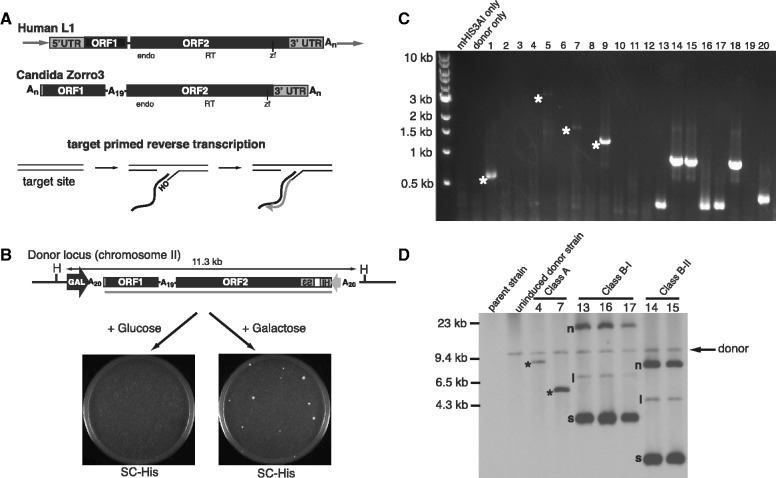
A novel class of LINE retrotransposition products. (**A**) Structure of L1 clade members and proposed mechanism of non-LTR retrotransposon replication. Top, comparison of the structures of human L1 and *Candida albicans* Zorro3. Both elements contain two ORFs and endonuclease (endo), reverse transcriptase (RT) and zinc finger (zf) domains. Genomic L1s are typically flanked by target site duplications (flanking arrows). Genomic Zorro3s are flanked by poly(A) tracts, which may include poly(A) target site duplications. Bottom, general mechanism for the initiation of non-LTR retrotransposon replication. (**B**) Assay to induce Zorro3 retrotransposition. A donor Zorro3 marked with a *HIS3AI* (47) retrotransposition cassette (Zorro3mHIS3AI) is present at the LYS2 locus of chromosome II. To induce retrotransposition, cells are grown on galactose and then plated on SC-His to select retrotransposition events. H = HaeII. Bar below Zorro3 represents the probe used for Southern blots in all subsequent figures. (**C**) Example of LM-PCR products. Each lane represents the LM-PCR product from an independent Zorro3 retrotransposition event. Owing to variable EcoRI distances from the Zorro3 3′ end, Class A LM-PCR products are not always amplified. ‘mHIS3AI only’ is an LM-PCR reaction from a strain containing only the mHIS3AI cassette. ‘Donor only’ is an LM-PCR reaction from a strain containing Zorro3mHIS3AI, but not a retrotransposition event. Class A products are noted with an asterisk. (**D**) Southern analysis of class A and class B events. Genomic DNA from the indicated clones was digested with HaeII and probed for Zorro3 sequence. Arrow indicates the band representing the original Zorro3mHIS3AI donor. * = class A insertions. n = nicked. l = linear. s = supercoiled.

Most available information on the biology of L1 clade members is derived from experiments with the mammalian L1 elements. An L1 element produces a bicistronic transcript encoding two proteins, ORF1 and ORF2 (Figure 1A). ORF1 protein binds to single-stranded nucleic acids with high affinity and possesses nucleic acid chaperone activity (21–25). ORF2 encodes endonuclease (26) and reverse transcriptase activities (27–30). All of these activities are important for retrotransposition (26,31–33). Complete mechanistic details of how LINE elements retrotranspose are unclear; however, a model of the initial steps of reverse transcription, called target-primed reverse transcription (TPRT), has emerged from biochemical experiments with the related R2 element of *Bombyx mori* (34). Later experiments with human L1 support this TPRT model (35), in which the L1 ribonucleoprotein particle (RNP), consisting of L1 RNA, ORF1 and ORF2, enters the nucleus and nicks a chromosomal target site. Reverse transcription ensues using L1 RNA as the template and the free 3′ end at the chromosomal nick as the primer (Figure 1A). Thus, L1 cDNA synthesis and chromosomal attachment is concurrent (in contrast to retroviral and LTR-retrotransposon cDNA synthesis). Completion of the insertion involves a second nick on the opposite DNA strand of the target site, which serves as the primer for plus strand DNA synthesis. Consistent with this mechanism, all characterized products of authentic L1 retrotransposition to date have been chromosomally integrated (26,33,36–40).

The *Candida albicans* genome contains an active yeast non-LTR retrotransposon called Zorro3 (41,42). Phylogenetic analysis of the Zorro3 reverse transcriptase domain places Zorro3 in the L1 clade (41). Genomic Zorro3 elements tend to be flanked by polyadenosine [poly(A)] tracts (Figure 1A). The poly(A) flanks are likely derived from three sources—the target site [Zorro3 tends to integrate into poly(A) tracts], a possible target site duplication [if both endonuclease nicks are within the target site poly(A) tract], and the mRNA poly(A) tail. As these sequences are identical homopolymers, we cannot unambiguously determine the source for all of the nucleosides in the poly(A) tracts. Another distinguishing feature between Zorro3 and mammalian L1 elements is that the former contains a type I ORF1, whereas the latter contains a type II ORF1 (43). Type I ORF1s contain CCHC zinc knuckle motifs instead of the conserved mammalian C-terminal domain, and are found in at least 5 different non-LTR retrotransposon clades (including non-mammalian members of the L1 clade), indicating their ancient origin (43). We previously redesigned and synthesized an *Saccharomyces cerevisiae*-compatible version of Zorro3 (40). We found that Zorro3 is able to retrotranspose in *S. cerevisiae* and maintains the requirement for ORF1 and ORF2 motifs. In addition, chromosomally integrated Zorro3 retrotransposition events revealed structural similarities to L1 integrations in human and mouse, suggesting that Zorro3 retrotransposition in *S. cerevisiae* is mechanistically similar to mammalian L1 retrotransposition. Here, we describe a previously unknown second class (class B) of retrotransposition products from Zorro3. Rather than being chromosomally integrated, class B products are RNA-derived DNA circles that represent the majority of retrotransposition products formed by Zorro3. This is the first time LINE elements have been shown to produce extrachromosomal products from the retrotransposition reaction. These episomal products may be a previously unrecognized side product or an intermediary source of active LINE retrotransposition.

## MATERIALS AND METHODS

### Yeast strains

The yeast strain used in Figures 1 through 3 (JHY 339) has been already described (40). The Zorro3pA^−^ strain, JHY343, was generated by converting wt Zorro3 to Zorro3pA^−^ by two-step gene replacement using pRS406-derived plasmids, as previously described (40). To test nucleic acid enzyme mutants for effects on class B frequency, we used the strain JHY869 to knock out compete open reading frames of desired genes by PCR-mediated disruption (44). JHY869 contains the Zorro3mHIS3AI and *His3Δ200* alleles backcrossed four times to YAD373 (45), to generate a retrotransposition strain with an s288c background with high sporulation frequency. Our laboratory has since switched to this strain for all Zorro3 retrotransposition studies because it is isogenic with the sequenced yeast genome and the yeast knockout collection, and it contains three alleles [RME1(ins-308 A), TAO3(E1493Q) and MKT1(D30G)] that allow it to sporulate with high efficiency for easier genetic manipulation. All yeast strains and genotypes are listed in Supplementary Table S1.

### Primers

All primers in this study are listed in Supplementary Table S2.

### Southern analysis

Genomic DNA was prepared as described previously (46). One microgram of genomic DNA was digested with the appropriate restriction enzyme, run on a 0.8% agarose gel, and blotted in 0.4 M NaOH to positively-charged nylon membrane (Millipore). Hybridizations were performed in Ultrahyb (Ambion) at 42°C. Riboprobes were biotin-16-UTP labelled T7 *in vitro* transcription products of JH328/JH329 PCR products (Zorro3mHIS3 probe) or CD15/CD16 PCR products (tubulin probe). Detection was performed with the Phototope-star detection kit (New England Biolabs).

### Class B product stability assays

A His^+^ colony from a SC-His plate was patched onto a yeast extract peptone dextrose (YPD) plate and incubated at 30°C for 24 hours. Cells from this YPD plate were used to inoculate a 2 mL YPD culture and incubated at 30°C for 24 h. Serial dilutions of this culture were plated on YPD plates, and colonies were allowed to form for 2–3 days at 30°C. Plates with ∼100–300 colonies were replica plated to both SC-His plates and new YPD plates. Percentage stability is defined as the number of colonies that grow on the SC-His plate divided by the number of colonies that grow on the YPD plate.

### Zorro3 retrotransposition assays and colony PCR

Quantitative Zorro3 retrotransposition assays were performed essentially as described previously (40). When isolating retrotransposition events to assay for class B products, induction of retrotransposition was performed by patching strains on SC+galactose plates (instead of cultures) to ensure that each His^+^ colony was an independent event. The induction plates were replica plated to SC-His to select for His^+^ events. Colony PCR (cPCR) was performed by inoculating 2.5 μL of H_2_O. This mixture was subjected to two freeze-thaw cycles, then used in a 20 μL PCR reaction containing 500 nM each primer, 200 nM dNTPs and 0.25 units ExTaq (Takara). JH774/JH775 amplifies class B-I, and JH774/JH776 amplifies class B-II. Conditions were 94°C for 4 min, 42 cycles of 94°C for 15 s, 56°C for 15 s, 72°C for 1 min, followed by a 2-min 72°C extension. Class B events were cloned into a standard TA vector for sequencing.

## RESULTS

### An unusual class of Zorro3 retrotransposition products

We used a previously described assay (Figure 1B) to detect Zorro3 retrotransposition events in budding yeast as His^+^ cells (40). This assay depends on the splicing of a synthetic intron out of a reverse reporter (47) placed in the Zorro3 3′ UTR. A functional HIS3 reporter is made only after Zorro3 transcription, splicing and reverse transcription/integration. Zorro3 induction produced cells that formed colonies of heterogeneous sizes on SC-His plates, with a large proportion of small colonies. Ligation-mediated PCR (LM-PCR) of 3′ flanking sequence from independently derived His^+^ clones revealed two main classes of products (schematic in Supplementary Figure S1). The first class was typically present in the biggest colonies and generated LM-PCR products of various sizes (e.g. Figure 1C, lanes 1, 5, 7, 9). This class represents chromosomally integrated retrotransposition events, which are described in detail elsewhere (40). We will henceforth refer to these classic, chromosomally integrated products as ‘class A’ products. The second class of retrotransposition events was present in some of the large colonies and almost all of the small colonies, and gave rise to LM-PCR products with characteristic band sizes of either ∼400 base pairs or ∼1000 base pairs (Figure 1C, lanes 13–18, 20). Sequencing revealed that the 3′ flanking sequence in these PCR products was either Zorro3 5′UTR/ORF1 or Zorro3 interORF/ORF2, respectively (Supplementary Figure S1). We classified these retrotransposition events as ‘class B’, and further distinguished them as ‘class B-I’ (3′ flanked by ORF1) or ‘class B-II’ (3′ flanked by ORF2).

Zorro3 has a preference to integrate into chromosomal poly(A) tracts (40,42). As the donor Zorro3 on chromosome II contains poly(A) tracts at the 5′ and interORF regions (Figure 1B), we hypothesized that class B products were simply integrants at these locations. To test this hypothesis, we performed Southern analysis on HaeII-digested genomic DNA from strains containing class A, B-I and B-II products (Figure 1D), using Zorro3mHIS3 sequence as a probe. As expected, class A products (clones 4 and 7) were integrated at a new site, generating a band distinct from the original donor band. We predicted that if class B products were integrated into the donor Zorro3, the original donor band would increase in size. However, the donor band was unaltered in class B strains, indicating that class B products are not integrated into the donor locus. Instead, class B products appeared as three new bands in addition to the donor band (Figure 1D, clones 13–17). Within each subclass (B-I or B-II), the banding pattern was virtually identical. Because the His^+^ clones used for Southern analysis were produced independently, this suggests that class B-I and B-II products are specific structures that are reproducibly generated on induction of Zorro3 retrotransposition. The three bands representing class B products bear a striking resemblance to the three forms of DNA (nicked, linear and supercoiled) often seen in plasmid preparations, and the intensity of the bands relative to the donor band suggests they are present in multiple copies. Thus, we next sought to investigate whether class B products were DNA circles.

### Class B products are DNA circles

**Figure 2.**
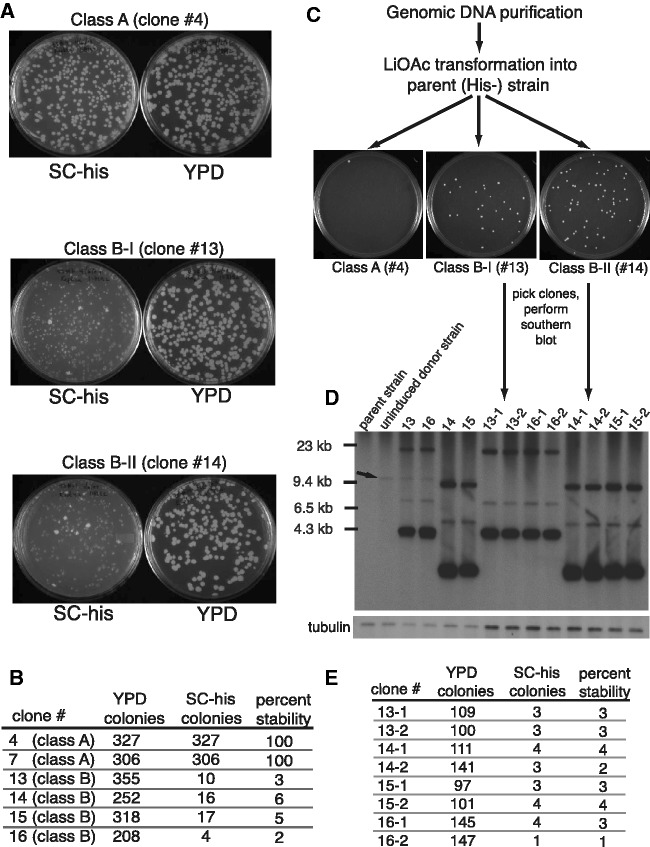
Instability and transfer of class B products between strains. (**A**) Representative examples of class A and class B product stability. (**B**) Quantitation of the experiment described in panel A. (**C**) Transfer of class B product between strains. Total genomic DNA purified from strains containing a class A or class B insertion was used to transform His^−^ yeast cells without pre-existing Zorro3 sequence. Transformations were plated on SC-His. His^+^ colonies represent successful maintenance of the retrotransposition product under selection. (**D**) Class B transformants harbour the class B episome. Genomic DNA from His^+^ transformants described in panel C was digested with HaeII and subjected to Southern analysis. The donor (arrow) and class B products are both present in the original class B strains (13–16), but only the class B product is transferred to the transformants (13-1, 13-2, etc.). (**E**) Class B products transferred to new strains exhibit instability. His^+^ stability testing described in panels A and B was repeated with class B transformants generated in panel C.

As class A products are integrated into a host chromosome, they are expected to be faithfully replicated and partitioned to daughter cells along with the rest of the chromosome. Episomes, on the other hand, may eventually be lost from a dividing population, unless they contain a centromere or other partitioning system. To test whether class B products are stably transmitted to daughter cells, we grew class B, His^+^ clones under non-selective conditions for 2 days. The resultant population quickly became predominantly His^−^ (Figure 2A and B). In contrast, class A clones remained 100% His^+^ after non-selective growth. These results are consistent with hypothesis that class B products are episomal circles.

We next tested the transformation ability of class B products. We transformed total genomic DNA preparations from class A or class B clones into a Zorro3^−^, His^−^ strain. We reasoned that for class A genomic DNA to convert this parental strain to His^+^, a fragment of transformed DNA containing the spliced *HIS3* gene would need to integrate into the host chromosome via homologous recombination or non-homologous end joining. Given that DNA fragments containing the *HIS3* gene are expected to be a small fraction of the total nucleic acid in a genomic DNA prep, transformation to His^+^ by class A DNA is a rare event (Figure 2C, left). However, transformation with the same amount of class B DNA led to comparatively high number of His^+^ colonies (Figure 2C, middle and right). This suggests that class B products are in the more easily transformable circular form. To confirm this, we isolated total genomic DNA from ‘recipient’ strains (e.g. 13-1, 13-2, etc.) newly transformed with class B DNA and probed for Zorro3 sequence (Figure 2D). The respective class B products were present in the recipient strains, but the chromosomally integrated donor from the original strains was not transferred (arrow in Figure 2D). The His^+^ phenotype exhibited the same instability shown in original class B strains (Figure 2E). These data are consistent with class B products being plasmids.

**Figure 3.**
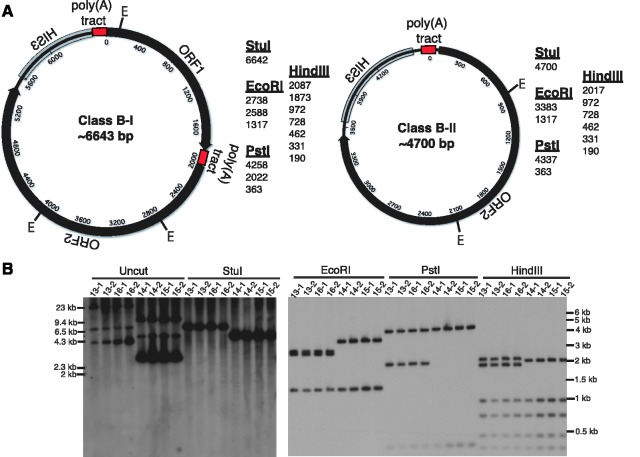
Structural characterization of class B products. (**A**) Predicted structures and restriction fragment sizes of class B-I and class B-II circles. (**B**) Verification of predicted class B structures. Genomic DNA from donorless strains containing a class B circle were digested with the indicated enzyme and subjected to Southern analysis with the Zorro3mHIS3 probe shown in Figure 1B.

As Zorro3 has three polyadenosine [poly(A)] tracts corresponding to the locations where class B circles are ‘joined’, we inferred the general structure of class B-I and class B-II forms (Figure 3A). We then confirmed these structures by restriction digests of genomic DNA from strains containing a class B-I or class B-II product (but not the original integrated donor) and probing for Zorro3 (Figure 3B). In all cases, we obtained the predicted restriction pattern, demonstrating unambiguously that non-LTR retrotransposition can, in some cases, result in circular products. The circular structure of these products also explains the size of the bands obtained from LM-PCR (shown in Supplementary Figure S2 schematic).

### Class B products can form with high frequency

**Figure 4.**
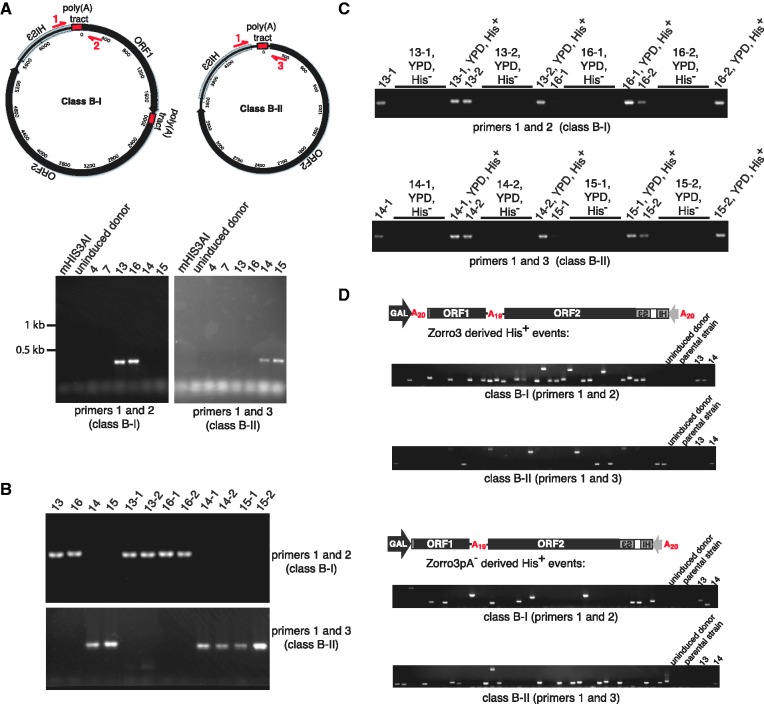
Class B products form with high frequency. (**A**) A cPCR assay to detect class B products. The indicated primers (in red) were used for colony PCR reactions to specifically amplify products in strains containing class B-I or class B-II circles. (**B**) The cPCR assay is sensitive and specific for class B products. Original class B strains and strains converted to His^+^ by class B genomic DNA transformation (Figure 2C and D) were subjected to the cPCR assay. (**C**) His^+^ phenotype tracks with the class B circle. A His^+^ stability experiment (described in Figure 2E) was performed on parent strains transformed with a class B product. Colonies that lost the His^+^ phenotype (YPD, His^−^) and rare colonies that retained the phenotype (YPD, His^+^) were tested by the cPCR assay for the presence of a class B episome. (**D**) Class B products constitute the majority of Zorro3 retrotransposition events. For two strains (containing Zorro3mHIS3AI or Zorro3pA^−^mHIS3AI), 44 independently derived retrotransposition events (all unlabelled lanes) were tested by cPCR for the presence of class B-I or class B-II products. 13 = an original class B-I clone (control). 14 = an original class B-II clone (control).

To simplify analysis of large numbers of retrotransposition events, we used a colony PCR (cPCR assay). Primers flanking the class B-I or class B-II junctions (primers 1 and 2 or primers 1 and 3 in Figure 4A) specifically detected the presence or absence of class B circles (Figure 4A–C). We used the cPCR assay to measure class B product frequencies. We induced 44 independent retrotransposition events. Remarkably, 24/44 clones contained a class B-I product, and 8/44 clones contained a class B-II product (Figure 4D, top). Thus class B retrotransposition can be a frequent event. As the formation of circles is possibly augmented by a template switch between the poly(A) tracts of the Zorro3 mRNA and the A-rich top strand of the Zorro3 target site, we investigated whether deleting the 5′ poly(A) tract of Zorro3 (Zorro3pA^−^) eliminated class B-I products. We found that 12/44 retrotransposition clones derived from Zorro3pA^−^ contained a class B-I event, suggesting that the long stretch of homology [in this case, poly(A)] is not required for circularization (Figure 4D, bottom).

### Pre-existing homology is not required for class B product formation

**Figure 5.**
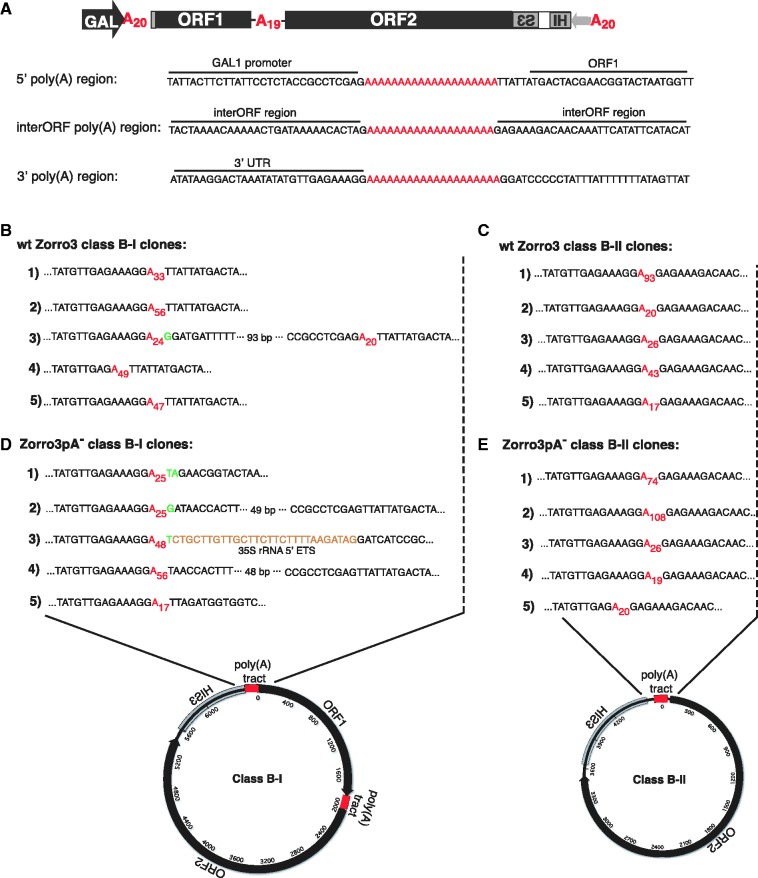
Pre-existing homology is not required for class B product formation. (**A**) Structure of Zorro3 donor and view of Zorro3 regions where most class B junctions occur. Class B-I circles typically form from a joining between the 3′ poly(A) region and the 5′ poly(A) region, whereas class B-II circles typically form from a joining between the 3′ poly(A) region and the interORF poly(A) region. Zorro3pA^−^ sequences are identical to Zorro3 except the stretch of 20 (A)s in the 5′ poly(A) region is deleted. (**B–E**) Sequences of cloned class B product junctions derived from wild-type Zorro3 or Zorro3pA^−^ donors. Untemplated nucleotides are indicated in green. Yeast genome sequences are indicated in brown.

To learn more about the nature of the junctions of class B circles (junction defined as the region corresponding to where the 5′ and 3′ ends of the retrotransposed element are joined to form the circle), we cloned and sequenced the junctions of five independent class B-I and class B-II clones derived from either wild-type Zorro3 or Zorro3pA^−^ (Figure 5A–E). Sequencing revealed that 3/5 wild-type class B-I clones (#1, 2, 5 from Figure 5B) comprised full-length Zorro3 sequence with the 3′ and 5′ ends joined by a poly(A) tract. The only difference between these clones was the length of the joining poly(A) tract. A fourth clone (#4) was nearly identical, but missing two Gs at the 3′ end before the poly(A) junction. This could be due to an alternative cleavage and polyadenylation site, or internal priming of Zorro3 mRNA during the initial step of reverse transcription (48). The remaining clone (#3) contained 113 base pairs of sequence upstream of the 5′ poly(A) tract, preceded by an untemplated G, suggesting that this clone was derived from an mRNA that initiated transcription upstream of the 5′ poly(A) tract.

The junctions of class B-II products derived from Zorro3 and Zorro3pA^−^ were essentially the same, with variations in poly(A) length being the only difference between clones (Figure 5C and E). One clone (Zorro3pA^−^ #5) was missing two Gs at the 3′ end, but otherwise identical to the rest. The similarities between Zorro3 and Zorro3pA^−^ class B-II clones were expected, as no modifications were made to the interORF poly(A) tract in Zorro3pA^−^.

The aforementioned data suggests that, in most cases, stretches of homology [poly(A) tracts] between the 5′and 3′ ends of the element serve as the preferred connecting junction for class B circles. As other notable LINE elements (e.g. human and mouse L1) do not contain pre-existing homology between the 5′ and 3′ ends, we analysed Zorro3pA^−^ class B-I products to ask whether the pre-existing 5′ and 3′ homology in Zorro3 was an absolute requirement for class B circle formation. When the 5′ poly(A) tract was deleted, class B-I products still formed, but with greater heterogeneity and non-templated nucleotides at the junction (Figure 5D). Non-templated nucleotides are similarly seen at mammalian L1 5′ junctions (36,37). It has been proposed that these non-templated nucleotides are added onto the 3′ end of the minus strand by reverse transcriptase, facilitating microhomology-mediated template switching (49). Two of the truncation points (clones #2, 4) are upstream of the beginning of the element [where the 5’ poly(A) tract would normally be]. This upstream sequence may represent transcription initiating upstream of the Zorro3 start. Two of the truncation points (clones #1, 5) are downstream in the element. The final clone 5′ truncates downstream in Zorro3 sequence, but template switches to 35S rRNA sequence before template switching to Zorro3 3′ sequence. In a prior analysis of class A clones, we saw similar cases of template switching to endogenous cellular sequences (40). There is no appreciable pre-existing homology at the junction in any of the Zorro3pA^−^ class B-I clones. In 3/5 cases, there were untemplated bases at the junction point before the template switch. This demonstrates that pre-existing element junction homology is not a requirement for the formation of class B products.

### Mutations affecting class B frequency and ratio

Zorro3 retrotransposition events (which, as shown earlier, are predominantly class B products) are dependent on conserved residues in ORF1, ORF2 endonuclease and ORF2 reverse transcriptase (40). Thus, it is likely that class B products are formed via a variation of TPRT. The endonuclease requirement, in particular, suggests that for class B circles, the reaction requires nicked chromosomal DNA as the initiating primer for reverse transcription. This would mean that even retrotransposition intermediates destined to become circles would be initially attached to a chromosome.

Based on this assumption, we explored how a non-LTR retrotransposition intermediate could be excised from a chromosome. TPRT predicts the formation of 3′ flaps on the top and bottom strands of a retrotransposition intermediate (see Figure 1A, bottom for an example of the bottom strand flap). Cleavage of both of these flaps by a cellular endonuclease(s) could lead to excision of the L1 intermediate. Previous work has shown that the XPF/ERCC1, a 3′ flap endonuclease involved in nucleotide excision repair and homologous recombination, can restrict human L1 retrotransposition (50). This suggests a potential interaction between L1 intermediates and 3′ flap endonucleases.

**Figure 6.**
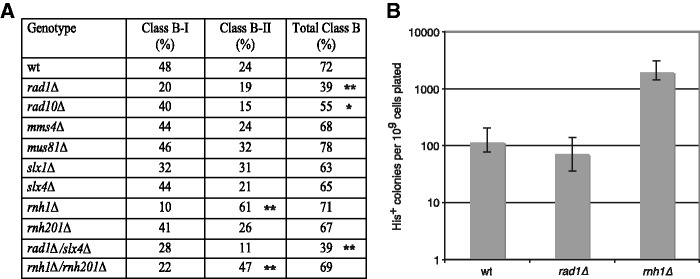
Mutations affecting class B frequency and ratio. (**A**) Zorro3 was induced for retrotransposition in the indicated mutants. In all, 94 independently derived clones were picked for each mutant and assayed by cPCR for the presence of Class B products. **P < 0.001. *P < 0.05. (**B**) Loss of *RNH1* leads to an increase in total Zorro3 retrotransposition. A quantitative retrotransposition assay was performed on the indicated strains. Frequency is presented as the average of five independent cultures. Black bars indicate the range of frequencies obtained.

To test whether the loss of 3′ flap endonucleases in yeast effects the formation of class B products, we knocked-out various genes involved in recognition and cleavage of branched nucleic acid structures. Rad1/Rad10 is the yeast ortholog of the mammalian XPF/ERCC1 heterodimer mentioned earlier. Mus81/Mms4 is an endonuclease involved in Holiday junction resolution with a preference for 3′ flap substrates (51,52). Slx4 is thought to recognize DNA structures and serves as a scaffold to recruit endonucleases, such as Rad1/Rad10, Mus81/Mms4 and Slx1 (a 5′ flap endonuclease) (53). In each of these mutant strains, we induced Zorro3 retrotransposition and assayed the percentage and ratio of class B events (Figure 6A). The most striking effects on formation of class B products were found in *rad1Δ* and *rad10Δ* strains, which showed a significant decrease in total percentage of class B products (*P* = 9 × 10^−^^6^ and *P* = 0.022, respectively; Fisher’s exact test). Although Slx4 can form a complex with Rad1/Rad10, deletion of *SLX4* did not lead to dramatic changes in class B frequency. In addition, a *rad1Δ/slx4Δ* double mutant showed a similar phenotype as the *rad1Δ* single mutant. Together, these data suggest that Rad1/Rad10 can act independently of Slx4. Although the percentage of class B product formation decreased in *rad1Δ* mutants, overall levels of retrotransposition remained largely unchanged (Figure 6B). Our data suggest that Rad1/Rad10 may play a role flap in cleavage during class B circle release from the chromosome.

Although LINE reverse transcription is usually initiated by endonuclease mediated nicks, in some cases, DNA breaks formed by other means can serve as a primer for reverse transcription (54,55). We sought to genetically induce DNA breaks by deleting RNaseH. *rnh1Δ* strains are expected to lead to a relative persistence of RNA/DNA hybrids. Multiple studies suggest that persistent RNA/DNA hybrids lead to DNA breaks (56–59). We predicted that an environment with preexisting DNA breaks would provide more potential chromosomal primers for LINE minus and/or plus strand synthesis. In the case of Zorro3, which has a low rate of retrotransposition (∼1 × 10^−^^6^ under the conditions of our assay), this may lead to an increase in retrotransposition frequency. Indeed, we found that in an *rnh1Δ* strain, overall Zorro3 retrotransposition frequency increases by greater than 10-fold (Figure 6B). Importantly, class B products still comprised ∼70% of these *rnh1Δ* retrotransposition events, indicating that class A and class B products are equally increased by introducing more potential chromosomal primers. This further strengthens our hypothesis that class B products are initiated at the chromosome.

At some point during TPRT, cleavage of the top strand must occur. The newly synthesized minus strand can then jump templates from retrotransposon RNA to the newly cleaved top strand. We hypothesized that in an *rnh1Δ* strain, the abundant availability of chromosomal breaks to serve as a primer (especially if a break is made at the target site) could allow premature minus strand template jumping and plus strand synthesis. Earlier minus strand template jumping [e.g. from the interORF poly(A) tract instead of the 5′ UTR poly(A) tract] would be expected to give rise to a greater proportion of class B-II products. Consistent with this hypothesis, in *rnh1Δ* strains, we observe a dramatic shift in the ratio of class B-I to class B-II products. Wild-type class B products are 33% class B-II, whereas *rnh1Δ* class B products are 85% class B-II (*P* = 3 × 10^−^^10^; Fisher’s exact test). Again, these results are consistent with minus and plus strand synthesis being initiated from the chromosome.

## DISCUSSION

We have shown that in addition to the expected integrated retrotransposition events, a LINE element can also generate episomal retrotransposition events. Previously, circular products derived from retroviruses/LTR-retrotransposons have been observed (60–62). However, retroviruses/LTR-retrotransposons replicate in a fundamentally different manner when compared with non-LTR retrotransposons. Retroviruses/LTR-retrotransposons complete linear cDNA synthesis in the cytoplasm. The formation of retroviral/LTR-retrotransposon circles is postulated to occur by various mechanisms, including homologous recombination between the 5′ and 3′ LTRs, non-homologous end joining of the linear cDNA ends, errors in reverse transcription and auto-integration (63–69). As these mechanisms involve retroviral intermediates, they seem unlikely to explain the formation of LINE circles. A less-specific class of circular DNA, small dispersed circular DNA (spcDNA), has been found to be present in essentially all mammalian cells (70,71). spcDNA consists of heterogeneous circles of sizes from hundreds to thousands of base pairs, and spcDNA is increased in cells with genome instability (70,71). spcDNA appears to be derived from chromosomal DNA and contains unique sequence, non-coding sequence and repetitive elements (including Alus and L1s). As short interspersed nuclear elements (SINEs) and LINEs are among the most abundant sequences in the genome, it is not surprising that circularized chromosomal fragments would contain these elements owing to their sheer percentage of genome mass. There is a variety of proposed mechanisms for the formation of spcDNA (72), the most likely being ‘pop-out’ of existing genomic DNA. Thus, the work described here is the first example of LINE circle formation via *bona fide* retrotransposition.

**Figure 7.**
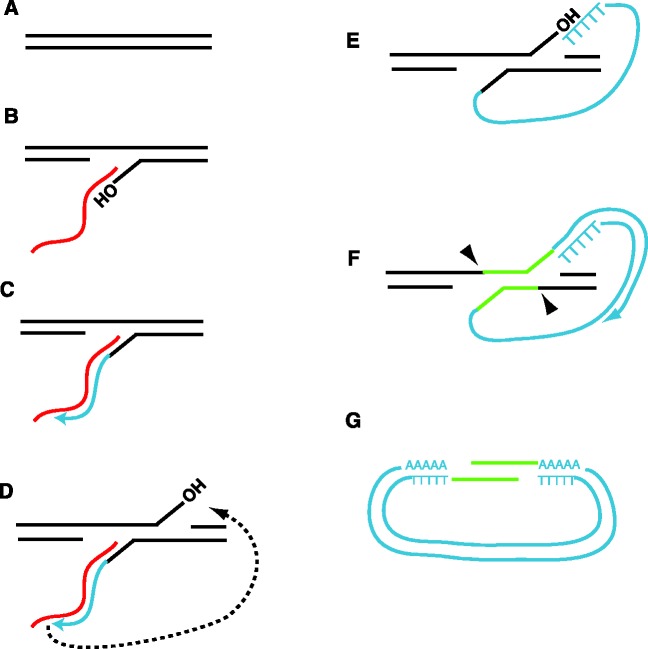
A speculative model for the formation of class B products. (**A**) Genomic target site. (**B**) Bottom chromosome strand nick and LINE mRNA annealing. (**C**) Minus strand synthesis. (**D–E**) Top strand chromosomal nick and template jump to top strand. (**F**) Recleavage of top and bottom strands (at arrowheads) to release the retrotransposition intermediate. The timing and placement of recleavages do not necessarily need to occur as indicated in the figure. Chromosomal target site DNA between the sites of recleavage have been highlighted in green for emphasis. As Zorro3 integrates into poly(A) tracts, the DNA highlighted in green would likely be poly(A) sequence. For other LINE elements, the DNA in green would likely correspond to part of the target site duplication. (**G**) Resolution of the excised circle by annealing of complementary strands. Red lines indicate RNA. Blue lines indicate newly synthesized retrotransposon DNA.

How are class B products formed? As mentioned earlier, class B products are normally endo-dependent. This suggests that chromosomal nicks are used as primers for reverse transcription, and thus class B products initiate attached to a chromosome. The requirement of ORF1, ORF2 endo and ORF2 reverse transcriptase strongly suggests that class B products are formed by TPRT, with a variation in retrotransposition intermediate resolution. The massive increase in both class A and class B retrotransposition in *rnh1Δ* strains further supports a TPRT-based model for class B formation, as *rnh1Δ* strains are expected to contain excess DNA breaks, a substrate for TPRT (54–59). Based on our data, we present a speculative model for circle formation in Figure 7. The steps in Figure 7A–E are identical to a standard TPRT reaction. We speculate that a structure-specific endonuclease can cleave the top and bottom strands in the vicinity of the 3′ flaps. As deletion of the Rad1/Rad10 3′ flap endonuclease significantly reduces class B product formation, Rad1/Rad10 may play a role in such a cleavage. However, Rad1/Rad10 cannot be solely responsible for this step, as loss of Rad1/Rad10 does not lead to the complete elimination of class B products. It is possible that other cellular nucleases play a redundant role, or even that the Zorro3 endonuclease may perform the recleavages. Further work is needed to distinguish between these possibilities. Once cleaved from the chromosome, the ends of the predicted linear excision product may contain genomic DNA corresponding to the genomic target site (highlighted in green in Figure 7F and G). These ends could anneal and be repaired by DNA repair pathways to form a circle. As Zorro3 targets poly(A) tracts, the highlighted green sequence in Figure 7F and G is likely to be poly(A), giving the appearance of a pure poly(A) junction. If this model were extrapolated to other LINE elements, the highlighted green segment of DNA would correspond to part of the target site duplication, and the size would be dependent on the distance between the top and bottom strand recleavages.

An obvious alternative to the model in Figure 7 is the ‘pop-out’ of stably integrated Zorro3 retrotransposition products owing to homologous recombination between the flanking poly(A) tracts. Several lines of evidence argue against this possibility. First, class B products were initially observed in *rad52Δ* strains, which are deficient in homologous recombination (Figures 1–5). Second, the portion of retrotransposition products that are class B is similar between a *rad52Δ* strain (Figure 4D) and Rad52^+^ strain (Figure 6A, wt). Third, we do not see clones that contain mixtures of class A/class B events or class B-I/class B-II events. We also have never witnessed the conversion of class A to class B products, or conversion of class B-I to the smaller class B-II products. This indicates that these retrotransposition products are independently generated and not derived from each other.

We do not currently know whether class B products can be formed by other L1 clade members. The majority of investigation on the L1 clade has focused on human and mouse L1, and thus far circular retrotransposition products have not been observed for these elements. However, the current assay for mammalian retrotransposition is not designed to detect circular retrotransposition products. Generally, unless a plasmid is specifically engineered to be maintained as an episome, clones derived from mammalian plasmid transfection and selection are the result of plasmid integration into the chromosome. Thus, if class B products are formed during mammalian retrotransposition assays, they are likely lost or integrated into the chromosome under the current assay schemes. We detected class B products in yeast owing to the fortuitous ability for Zorro3 class B products to be maintained in yeast under selection. This suggests that some subset of Zorro3 sequence can act as an autonomous replicating sequence in yeast. The ability for foreign sequence to replicate in yeast is not unprecedented (73), and we view the Zorro3 autonomous replicating sequence activity as serendipity. Even if other L1 clade members form class B products in their native hosts, we do not necessarily expect these products to contain an origin of replication.

Although we would expect any particular non-LTR retrotransposition-derived circle to exist only transiently, class B products may not necessarily be ‘dead ends’, as some LINEs (notably human and mouse L1) encode a promoter on their mRNA. Thus, if a mammalian full-length class B product is formed in a cell, this circle could in principle be a source for future retrotransposition events. As the circles would be newly synthesized DNA divorced from the chromosomal context, they may escape silencing and could be even more retrotranspositionally active (on a per copy basis) than chromosomal copies. This would be an example of how a transposon, in this case, a LINE element, could circumvent apparent ‘death by excision’, to potentially jump again. Further studies are needed to assess whether class B products can be formed in other organisms, especially in evolutionarily relevant cellular niches (e.g. the germ line of mammals).

## SUPPLEMENTARY DATA

Supplementary Data are available at NAR Online: Supplementary Tables 1–2 and Supplementary Figures 1–2.

## FUNDING

National Institutes of Health [R01GM090192]; the Carnegie Institution for Science. Funding for open access charge: Carnegie Institution for Science.

*Conflict of interest statement*. None declared.

## Supplementary Material

Supplementary Data
