# Supplementary material for: Circular retrotransposition products generated by a LINE retrotransposon
Source: Nucleic Acids Res. 2012 Sep 12;40(21):10866–77. doi: 10.1093/nar/gks859 (PMC3510499; doi:10.1093/nar/gks859)
Supplement: Supplementary Data [file supp_40_21_10866__index.html]

Circular retrotransposition products generated by a LINE retrotransposon — Circular retrotransposition products generated by a LINE retrotransposon — Supplementary Data 

# Circular retrotransposition products generated by a LINE retrotransposon

## Supplementary Data

files

**Files in this Data Supplement:**

- Supplementary Data - pdf file
