# Supplementary material for: Circular retrotransposition products generated by a LINE retrotransposon
Source: Nucleic Acids Res. 2012 Sep 12;40(21):10866–77. doi: 10.1093/nar/gks859 (PMC3510499; doi:10.1093/nar/gks859)
Supplement: Supplementary Data [file supp_gks859_nar-01845-a-2012-File009.pdf]

SUPPLEMENTARY INFORMATION

Fig. S1. Schematic of LM-PCR and classes of products obtained. LM-PCR was performed as described previously (40). This particular schematic shows a full-length insertions, but would be similar for short insertions. Linker length is not to scale.

Fig. S2. LM-PCR products formed from class B circles. E = EcoRI.

Table S1. List of yeast strains used in this study.

Table S2. List of primers used in this study.

Figure S1

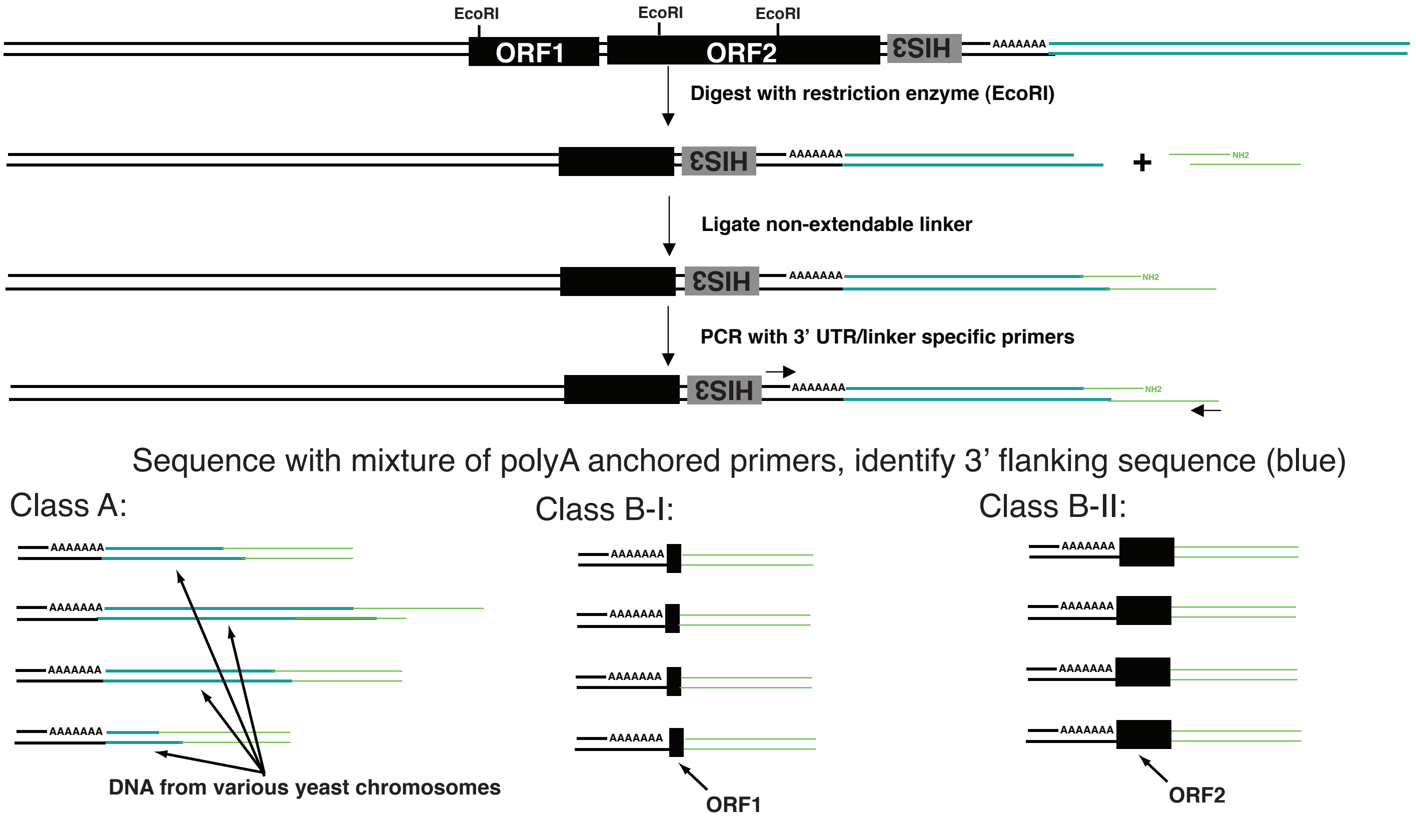

Fig. S1. Schematic of LM-PCR and classes of products obtained. LM-PCR was performed as described previously (40). This particular schematic shows a full-length insertion, but would be similar for short insertions. Linker length is not to scale.

Figure S2

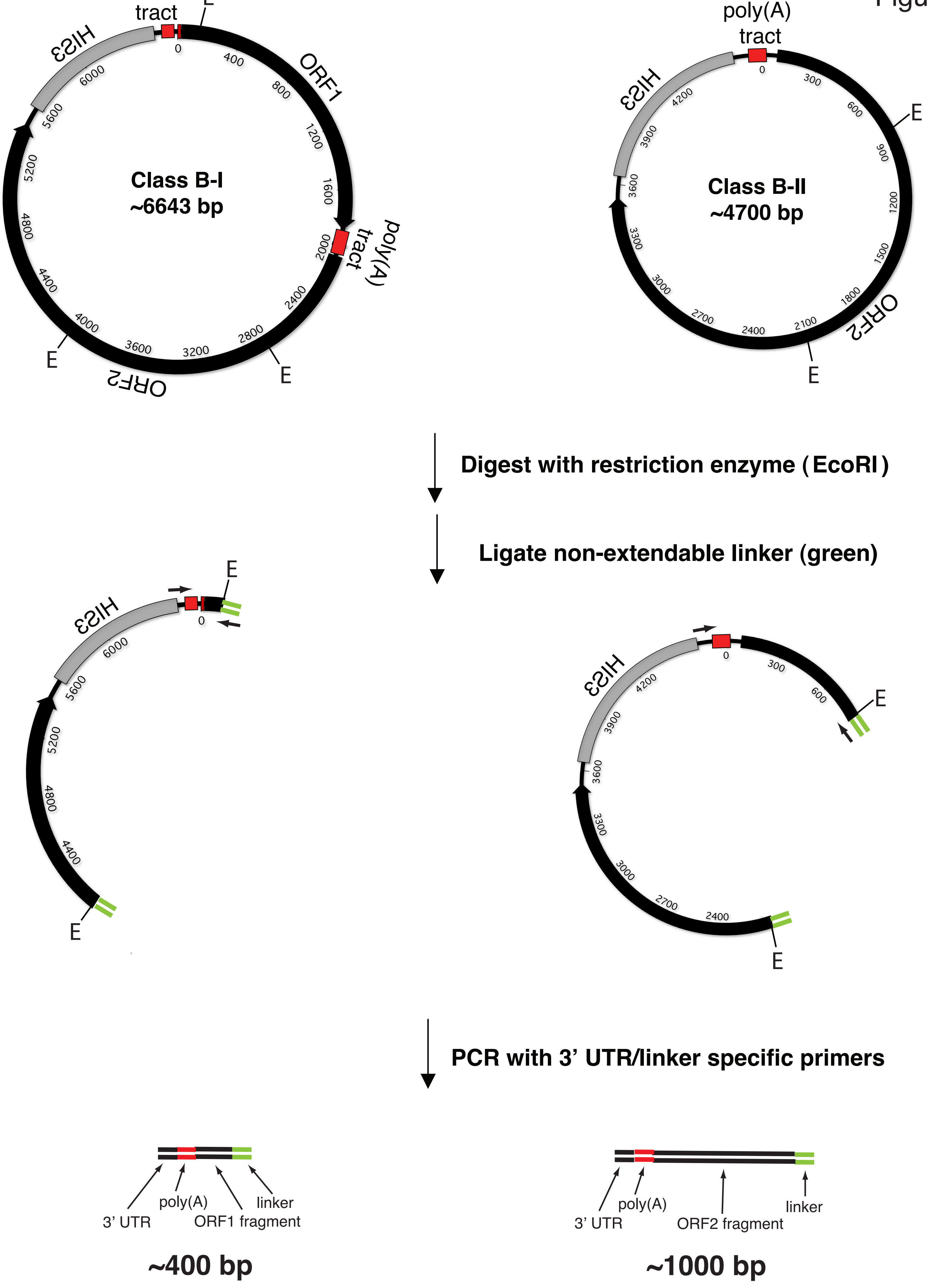

Fig. S2. LM-PCR products formed from class B circles. E = EcoRI.

Table S1
Yeast Strains used in this study

Strain	Genotype	Comment	Reference
GRF167	MATα his3Δ200 ura3-167 GAL+	Parent strain of JHY335, JHY339, JHY343	Boeke et al (1985)
JHY335	MATα his3Δ200 ura3-167 GAL+ lys2Δ::mHIS3AI rad52Δ::hygro spt3Δ::KanMX	"mHIS3AI" only	Dong et al (2009)
JHY339	MATα his3Δ200 ura3-167 GAL+ lys2Δ::Zorro3mHIS3AI rad52Δ::hygro spt3Δ::KanMX	wt Zorro3mHIS3AI strain	Dong et al (2009)
JHY343	MATα his3Δ200 ura3-167 GAL+ lys2Δ::Zorro3pA$^-$mHIS3AI rad52Δ::hygro spt3Δ::KanMX	Zorro3pA$^-$mHIS3AI strain	This study
JHY868	MATα his3Δ200 leu2Δ0 ura3Δ0 lys2Δ::Gal1Zorro3mHIS3AI RME1(ins-308A) TAO3(E1493Q) MKT1(D30G)	Figure 6B "wt"	This study
JHY881	MATα his3Δ200 leu2Δ0 ura3Δ0 lys2Δ::Gal1Zorro3mHIS3AI rad1Δ::URA3 RME1(ins-308A) TAO3(E1493Q) MKT1(D30G)	Figure 6B "rad1Δ"	This study
JHY885	MATα his3Δ200 leu2Δ0 ura3Δ0 lys2Δ::Gal1Zorro3mHIS3AI rad10Δ::URA3 RME1(ins-308A) TAO3(E1493Q) MKT1(D30G)	Figure 6B "rad10Δ"	This study
JHY889	MATα his3Δ200 leu2Δ0 ura3Δ0 lys2Δ::Gal1Zorro3mHIS3AI mus81Δ::URA3 RME1(ins-308A) TAO3(E1493Q) MKT1(D30G)	Figure 6B "mus81Δ"	This study
JHY893	MATα his3Δ200 leu2Δ0 ura3Δ0 lys2Δ::Gal1Zorro3mHIS3AI mms4Δ::URA3 RME1(ins-308A) TAO3(E1493Q) MKT1(D30G)	Figure 6B "mms4Δ"	This study
JHY897	MATα his3Δ200 leu2Δ0 ura3Δ0 lys2Δ::Gal1Zorro3mHIS3AI slx1Δ:URA3 RME1(ins-308A) TAO3(E1493Q) MKT1(D30G)	Figure 6B "slx1Δ"	This study
JHY901	MATα his3Δ200 leu2Δ0 ura3Δ0 lys2Δ::Gal1Zorro3mHIS3AI slx4Δ::URA3 RME1(ins-308A) TAO3(E1493Q) MKT1(D30G)	Figure 6B "slx4Δ"	This study
JHY905	MATα his3Δ200 leu2Δ0 ura3Δ0 lys2Δ::Gal1Zorro3mHIS3AI rnh1Δ::URA3 RME1(ins-308A) TAO3(E1493Q) MKT1(D30G)	Figure 6B "rnh1Δ"	This study
JHY909	MATα his3Δ200 leu2Δ0 ura3Δ0 lys2Δ::Gal1Zorro3mHIS3AI rnh201Δ::URA3 RME1(ins-308A) TAO3(E1493Q) MKT1(D30G)	Figure 6B "rnh201Δ"	This study
JHY924	MATα his3Δ200 leu2Δ0 ura3Δ0 lys2Δ::Gal1Zorro3mHIS3AI rad1Δ::URA3 slx4Δ::URA3 RME1(ins-308A) TAO3(E1493Q) MKT1(D30G)	Figure 6B "rad1Δ/slx4Δ"	This study
JHY928	MATα his3Δ200 leu2Δ0 ura3Δ0 lys2Δ::Gal1Zorro3mHIS3AI rnh1Δ::URA3 rnh201Δ::URA3 RME1(ins-308A) TAO3(E1493Q) MKT1(D30G)	Figure 6B "rnh1Δ/rnh201Δ"	This study

Table S2
Oligonucleotides used in this study

Oligo	Sequence	Comment
JH950	ttaagcggccgcgaattcCTAGAGCTCGCTGATCAGCCTCGA	BGHp(A) signal cloning
JH951	ttaagcggccgccaattgTCCCCAGCATGCCTGCTATTGTCTT	BGHp(A) signal cloning
JH952	ttaagcggccgcGTTGACATTGATTATTGACTAGTTATTAATAGTAATCA	CMV promoter cloning
JH953	ttaagcggccgcttaattaaGAGCTCTGCTTATATAGACCTCCCA	CMV promoter cloning
JH954	ttaagcggccgcggatccCCTTTATGTGTAACTCTTGGCTGAAGCT	EBV oriP cloning
JH955	ttaagcggccgcgaattcGCAGGAAAAGGACAAGCAGCGAAAATTCA	EBV oriP cloning
JH976	ttaagcggccgcagatctAAACGACCCAACACCCGTGCGTTTTATT	SV40neo with intron cloning
JH977	ttaagcggccgcGGATCCGGCTTACGGTATCGATCAGCTGTGGA	SV40neo with intron cloning
JH774	GATTGTAACCCCCTCGTATGTTTAT	class B cPCR primer
JH775	CACCTTTATCAGCAAAATCAAAACCA	class B cPCR primer
JH776	TAGGATTCATTTCTCTTCATTCACAGT	class B cPCR primer
JH944	TGCTAAATGTGTAAAAATAATATTGCACTATCCTGTTGAAAATATcaccataccacagcttttcaattca	RAD1 KO primer
JH945	TCGCATTTTATACTGATGTTTTAACAGGGTTCGTTAAATTAAACAcaccgcatagggtaataactgatat	RAD1 KO primer
JH843	ACTTATGAGACAGCCACGCAACACAAAAAAGGGCATAAACAAAGTcaccataccacagcttttcaattca	RAD10 KO primer
JH844	AGGATGGTAATAAGCATGGAACAGATTTATTAAAAGAAAATAGGAcaccgcatagggtaataactgatat	RAD10 KO primer
JH845	TTTCAAAGGATTGATACGAACACACATTCCTAGCATGAAAGCATcaccataccacagcttttcaattca	MUS81 KO primer
JH846	TTTCTTTATAAAACCTTGCAGGGATGACTATATTTCAAATTGCTAcaccgcatagggtaataactgatat	MUS81 KO primer
JH946	ATGGTATAGAATAATAGTAGTCACATATTGCAGCTAGTTAAATGAcaccataccacagcttttcaattca	MMS4 KO primer
JH947	CTGCCTTAAGGTATGTTCTTATATACAAAGTTTCGTTCGATCATCcaccgcatagggtaataactgatat	MMS4 KO primer
JH847	TTCTCTTTTTTCAAAAAATATTAAAAAGATAAGAAAGGTACCATcaccataccacagcttttcaattca	SLX1 KO primer
JH848	CATTATCTATAGAAAAAAAAGAACCGAAAATTGTGTCAGGCCCTAcaccgcatagggtaataactgatat	SLX1 KO primer
JH849	GTTCCATAATAATAACCAGTAGTTCAGTTGGGGAACTTAATAATcaccataccacagcttttcaattca	SLX4 KO primer
JH850	TTTGTTTTGTTTTTGTTTTGTCAAATTCTCAATCATTCGGCACTAcaccgcatagggtaataactgatat	SLX4 KO primer
JH868	GTGTCACTCCTTGCTTATCGAAGGAACTATCGATTCCTAATTATcaccataccacagcttttcaattca	RNH1 KO primer
JH869	TAGCTATGAATAAAAGTAAAAATAAACACAATCAACTGTGGACGAcaccgcatagggtaataactgatat	RNH1 KO primer
JH870	AACCTTGAAAACAACTACTGCACACCAAATTGATACGATTAAATcaccataccacagcttttcaattca	RNH201 KO primer
JH871	AGTATTACATGAAGATATATAGTATGTGCAAACTGGAGGTGATCAcaccgcatagggtaataactgatat	RNH201 KO primer
JH328	TTATTATGACTACGAACGGTACTAATGG	primer to make Zorro3mHIS3 probe
JH329	taatacgactcactatagggagaCTCAACATATATTTAGTCCTTATATACAGGTATA	primer to make Zorro3mHIS3 probe
CD15	ATGAGAGAAGTTATTAGTATTAATGTCGGT	primer to make tubulin probe
CD16	taatacgactcactatagggagaTTAAAATTCCTCTTCCTCAGCGTATGA	primer to make tubulin probe
JH1012	CTTTGCATATGCCGCCACCT	nested PCR primer
JH1013	GGCGTGAATTTTCGCTGCTT	nested PCR primer
JH1014	GGCAGGATCTCCTGTCATCT	nested PCR primer
JH1015	GCTCCTGCCGAGAAAGTATC	nested PCR primer
JH1016	CGCCTTCTTGACGAGTTCTT	nested PCR primer
JH1017	GGGATCGGCAATAAAAAGAC	nested PCR primer
JH1018	CCTTGTCGCTGCTGTAGGT	nested PCR primer
JH1019	AGATCTTCTCCCAGGTGGTG	nested PCR primer
JH1020	TGAAGCGGTAGATCACCTTG	nested PCR primer
JH1021	ATGGCCATCTTCACGATGTT	nested PCR primer
JH1022	TAGAAGCTGTTGGGCAGGAT	nested PCR primer
JH1023	TTCTCGATGCTCTGGAACAG	nested PCR primer
JH1024	ATCTCGTTGTGCACCCAGTA	nested PCR primer
JH1025	TCTGGGTCAGGTTCTTGATG	nested PCR primer
JH1026	GTTCAGGCCGTTGATGTTC	nested PCR primer
JH1027	AGGATGGTGATGTGGCTGT	nested PCR primer
JH1028	CACTTCTCGGTGTTGGTGAT	nested PCR primer
JH1029	GATGCACTCCTCCAGGTTCT	nested PCR primer
